# Continuous dynamic sliding mode control strategy of PWM based voltage source inverter under load variations

**DOI:** 10.1371/journal.pone.0228636

**Published:** 2020-02-06

**Authors:** Waqas Anjum, Abdul Rashid Husain, Junaidi Abdul Aziz, M. Abbas Abbasi, Hasan Alqaraghuli

**Affiliations:** 1 School of Electrical Engineering, Universiti Teknologi Malaysia, Johor Bahru, Malaysia; 2 Department of Electronic Engineering, Faculty of Engineering, The Islamia University of Bahawalpur, Punjab, Pakistan; Beijing University of Posts and Telecommunications, CHINA

## Abstract

For closed-loop controlled DC-AC inverter system, the performance is highly influenced by load variations and online current measurement. Any variation in the load will introduce unwanted periodic error at the inverter output voltage. In addition, when the current sensor is in faulty condition, the current measurement will be imprecise and the designed feedback control law will be ineffective. In this paper, a sensorless continuous sliding mode control (SMC) scheme has been proposed to address these issues. The chattering effect due to the discontinuous switching nature of SMC has been attenuated by designing a novel boundary-based saturation function where the selection of the thickness of boundary is dependent to the PWM signal generation of the inverter. In order to remove the dependency on the current sensor, a particle swarm optimization(PSO) based modified observer is proposed to estimate the inductor current in which the observer gains are optimized using PSO by reducing the estimation errors cost function. The proposed dynamic smooth SMC algorithm has been simulated in MATLAB Simulink environment for 0.2-kVA DC-AC inverter and the results exhibit rapid dynamic response with a steady-state error of 0.4*V* peak-to-peak voltage under linear and nonlinear load perturbations. The total harmonic distortion (THD) is also reduced to 0.20% and 1.14% for linear and non-linear loads, respectively.

## 1 Introduction

Voltage source inverter (VSI) is widely employed in power generation systems [1], uninterruptible power supplies [1, 2], electric vehicles systems (EVS) [3], and renewable energy systems [4, 5] to supply power to the various types of loads. In these applications, accurate regulation of the inverter output voltage and current is significantly important. One of the major factors that deteriorates the inverter output performance in these applications is the occurrence of sudden load changes [3]. As in generation system and EVs, the inverter is subjected to immense load variations ranging from zero to full load conditions [6] which introduces a periodic error in the inverter output. Furthermore, when the inverter system is subjected to the non-linear loads like motors and rectifiers, they cripple the output voltage of the inverter which results in high THD [7] that may cause damage to the industrial systems. Another factor that seriously sways the performance of VSI is the condition of faulty current sensor [8]. The control algorithms that are developed based on this feedback of the current sensor will be affected if the sensor is faulty or malfunctioning [9–11]. Therefore, for inverter controller design, it is imperative to design a sensorless robust controller that can withstand a wide range of load variations including non-linear loads with minimized THD and reduced steady-state error.

Proportional integral derivative (PID) controllers are widely employed in power electronics applications due to its simple implementation, however, linear PID controllers are not able to attain good performance and accuracy while dealing with nonlinear inverter system [4, 9, 12]. The enhanced computational power of the recent hardware devices has enabled the implementation of advanced robust control algorithms such as, repetitive control [13], *H*_∞_ control [14], model predictive control [15, 16], fuzzy & neural network based control [12, 17] and sliding mode control [4, 5, 7, 18] to improve the control performance of DC-AC inverter system against unavoidable sudden load variations. Among these control methods, SMC is more favorable control algorithm for inverter system because of its inherent switching nature [2] that is compatible with VSI gate switching. SMC has superior reference tracking capability and exhibits robustness against load parameter perturbations, although it suffers from chattering effect due to the presence of discontinuous signum function in the control law [19]. Chattering not only increases the switching losses of the inverter system but also injects high frequency harmonics into the output [18]. To attenuate this chattering effect, a hysteresis-SMC approach has been presented in [20] where a continuous saturation function replaces the discontinuous signum function. However, in this method, there is a trade-off between chattering removal and robustness where large boundary thickness will reduce the chattering but the robustness towards the load variation is decreased [21]. This constraint can be resolved by using adaptive hysteresis band approach as proposed in [22, 23] in which boundary thickness is varied proportional to the sliding surfaces, however, the complexity of using an extra feedback loop decreases its merit. In [24], fuzzy-based smooth control law has been introduced in which the fuzzy system selects a thin boundary near the sliding surface to ensure the robustness of the system. This method is effective in chattering attenuation but an extra feed-forward path is required for continuous sliding surface monitoring and evaluation of fuzzy rules which increases the computational burden of the system. Thus, to make the control law smooth while retaining the robustness, proper selection of the boundary layer is a critical task.

Furthermore in order to implement state feedback SMC, both the inductor current and capacitor voltage measurements are obtained via sensor reading [2, 5]. However, to obtain precise measurement of the inductor current is a challenging task because of inherent high frequency oscillations. The presence of this wideband current sensor reduces the fault tolerance capability of the control loop [9] due to sensor performance degradation with time. The common solution to this problem is to replace the sensors with state estimator. Various estimation algorithms have been presented in the literature such as current emulator [9], sliding mode observer [25] luenberger observer [10], pole placement based observation [26], linear quadrature regulator (LQR) [27], extended state observer (ESO) [28], etc. The performance and accuracy of these estimation techniques also depend upon proper gain selection methods. As in sliding mode observer, the gain must be greater than the bound of uncertainties present in the system. Calculation of uncertainty bound is challenging, thus, an approximated high gain bound is arbitrarily selected that results in the amplification of noise and chattering. In luenberger observer, the gains are designed to be proportional to the poles of inverter system which cause instability due to the high imaginary part of system poles. Pole placement and LQR based estimation approaches provide robust estimated values but their gain matrix design procedure is heuristic in approach with constraints on system observability. ESO, however, can estimate the states even in the presence of uncertainties but, in order to reduce the estimation error, high gains values for ESO parameters are arbitrarily selected which compromises the noise tolerance capability [29] of the observer. Although observer-based approaches provide better accuracy and robustness, their gain design procedure however is a tedious and complicated task.

In this paper, a dynamic SMC for VSI control is proposed in order to deal with inverter load variations. A novel fixed frequency continuous control law has been designed using the PWM operating frequency requirements. This smoothed control law algorithm not only attenuates the chattering, which helps to keep THD at the minimum allowable limit, but also retains the robustness against load parameter variations. A modified state observer is introduced to reconstruct the inductor current that removes the dependency on the current sensor while the optimal observer gain selection has been performed using PSO. The effectiveness of the proposed algorithm is verified through MATLAB simulations where the system is subjected to both linear and nonlinear load changes. The noteworthy contributions of the paper are as follows:

- The proposed controller offers a simple and accurate remedy for reference tracking and THD minimization with only one design parameter.
- The observer proposed provides the robust estimates of the inductor current and exhibits stable response against step load variations where the traditional observer become unstable.
- The complexity of observer gain design has been significantly reduced by using PSO technique.
- A novel boundary layer containing PWM switching characteristics and LC filter components values has been proposed, which is not only simple and robust but also reduces the chattering phenomenon to a significant level.

The rest of the paper is organized as follows: Inverter modeling is given in section 2. Section 3 deals with the designing of smooth dynamic control law. Section 4 is devoted to stability analysis of the proposed algorithm. Observer gain optimization using PSO is described in section 5 followed by simulation results and discussions in section 6. Finally, the paper is concluded in section 7.

## 2 Inverter modeling

**Fig 1:**
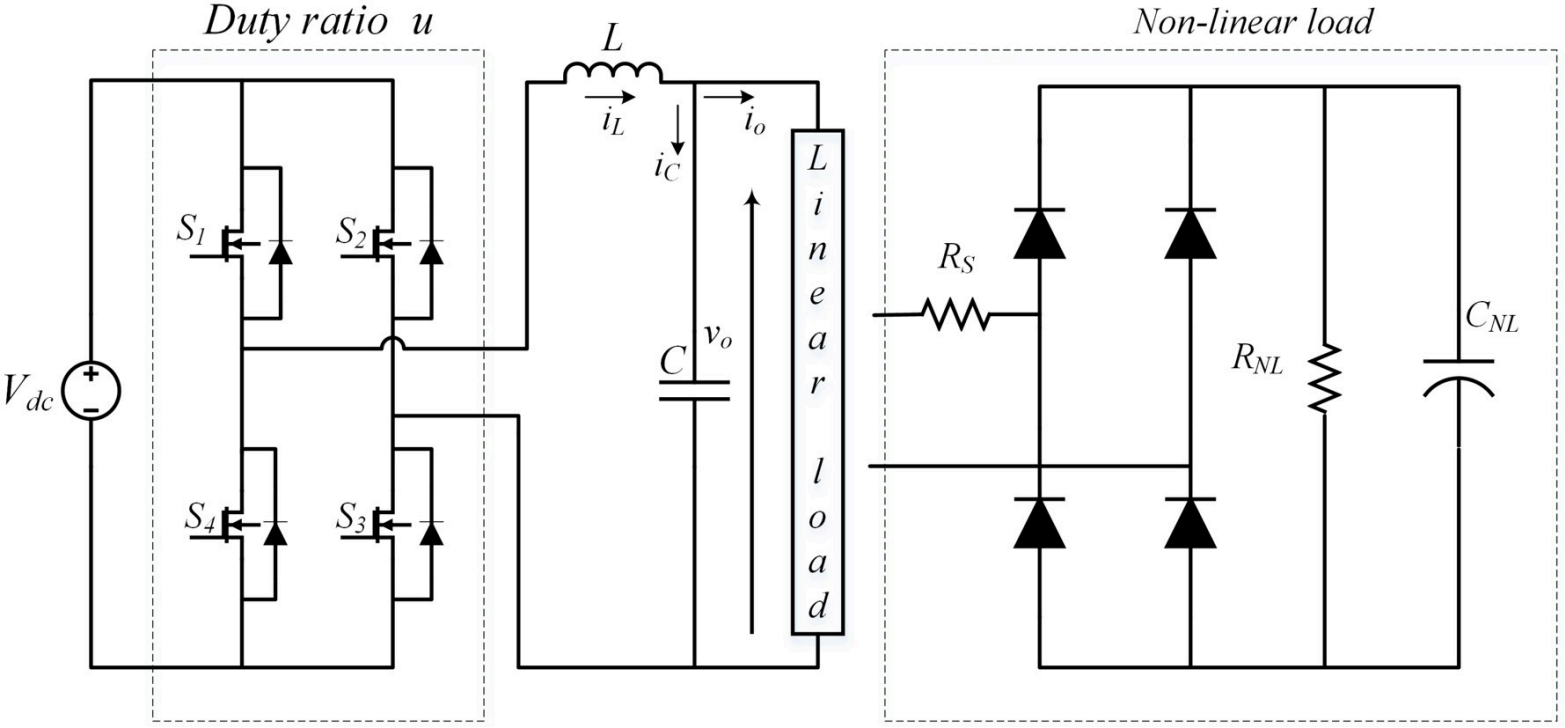
Single phase DC-AC inverter.

A single-phase inverter is shown in Fig 1, where *V*_*dc*_ is the *DC* input voltage, *L* is the inductor and *C* is the capacitor that forms the low pass filter. *S*_1_ → *S*_4_ are the semiconductor switches comprised of IGBTs, that change their states in a complimentary manner i.e. both the switches in a leg cannot turn on at the same time. These switching states are controlled by the PWM signal that contains a set of binary values {0, 1} based on controller output *u*, as described in (1). *i*_*L*_, *i*_*C*_ and *i*_*o*_ are the inductor current, capacitor current and output current, respectively. Output voltage *v*_*o*_ is taken across the filter capacitor which follows the desired sinusoidal reference voltage.

$$\begin{array}{c} u=\{\begin{array}{c} 1\;S_{1}\&S_{3}=on\;S_{2}\&S_{4}=off\\ 0\;S_{1}\&S_{3}=off\;S_{2}\&S_{4}=on \end{array} \end{array}$$ (1)

**Fig 2:**
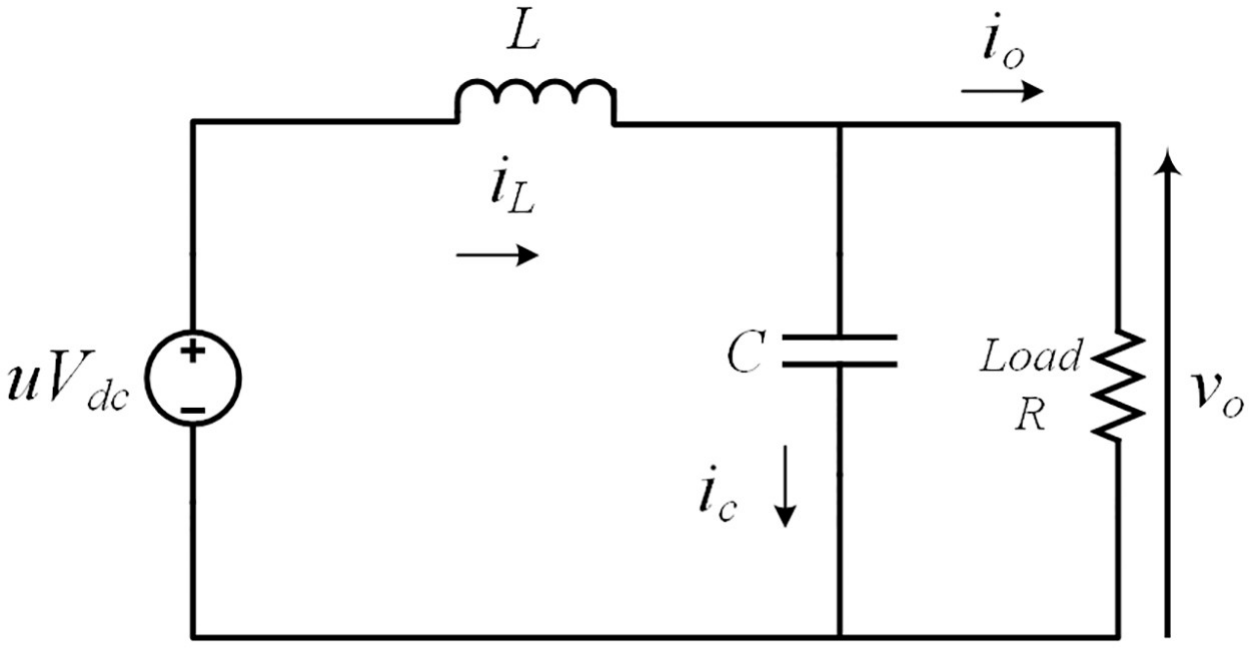
Equivalent circuit.

In both the cases the output of the inverter that serve as an input to the *LC* filter is *uV*_*dc*_. The equivalent circuit can be represented as in Fig 2 where, *R* is the resistive load and $i_{o}=\frac{v_{o}}{R}$ is the current through that load. The inverter dynamics by using Kirchhoff’s laws can be expressed as

$$\begin{array}{c} \left\{ \begin{array}{c} {\dot{v}}_{o}=\frac{i_{L}}{C}-\frac{v_{o}}{RC}\\ {\dot{i}}_{L}=\frac{uV_{dc}}{L}-\frac{v_{o}}{L} \end{array}\right. \end{array}$$ (2)

For this system, the output voltage *v*_*o*_ and inductor current *i*_*L*_ are considered as state variables, where output voltage *v*_*o*_ is designed such that it is able to track the desired sinusoidal reference voltage *v*_*ref*_ even if the system is subjected to various linear and non-linear loads. From Eq (2) it is clear that the load is coupled to the output voltage *v*_*o*_ and inductor current *i*_*L*_ where any variation in the load will affect both of the state variables. Thus, the main control objective is to develop a robust controller for this class of VSI system that can track the desired reference sinusoidal voltage with minimized steady state error and THD even the system is subjected to load variations.

## 3 Sliding mode controller design

The SMC is well known for its robust tracking capabilities. The SMC control law is designed in two steps; first, the sliding surface is designed for desired system dynamics and second, a switching control law is developed that forces the system states to meet the reaching condition. The conventional SMC control law can be defined as

$$\begin{array}{c} u=sign(s(x))=\{\begin{array}{c} +1\;if\;s(x)\geq 0\\ -1\;if\;s(x)<0 \end{array} \end{array}$$ (3)

where *s*(*x*) is termed as sliding surface. The control law in (3) is a switching function that indicates the controller output depends on the sliding surface function. The inverter system presented in Fig 2 can be considered as second order system, thus, the sliding surface can be written as

$$\begin{array}{c} s\left(x\right)=(\frac{d}{dt}+\lambda )x_{1}={\dot{x}}_{1}+\lambda x_{1} \end{array}$$ (4)

where *x*_1_ and *ẋ*_1_ are tracking error and its derivative, respectively, defined as

$$\begin{array}{c} \left\{ \begin{array}{c} x_{1}=v_{ref}-v_{o}\\ x_{2}={\dot{x}}_{1}={\dot{v}}_{ref}-{\dot{v}}_{o} \end{array}\right. \end{array}$$ (5)

where *v*_*ref*_ is the desired reference voltage for the inverter. By inserting (2) into (5), *x*_2_ will become

$$\begin{array}{c} x_{2}=-\frac{i_{L}}{C}+\frac{v_{o}}{RC}+{\dot{v}}_{ref} \end{array}$$ (6)

while the sliding surface can be written as

$$\begin{array}{c} s\left(x\right)=\lambda \left(v_{ref}-v_{o}\right)-\frac{i_{L}}{C}+\frac{v_{o}}{RC}+{\dot{v}}_{ref} \end{array}$$ (7)

**Fig 3:**
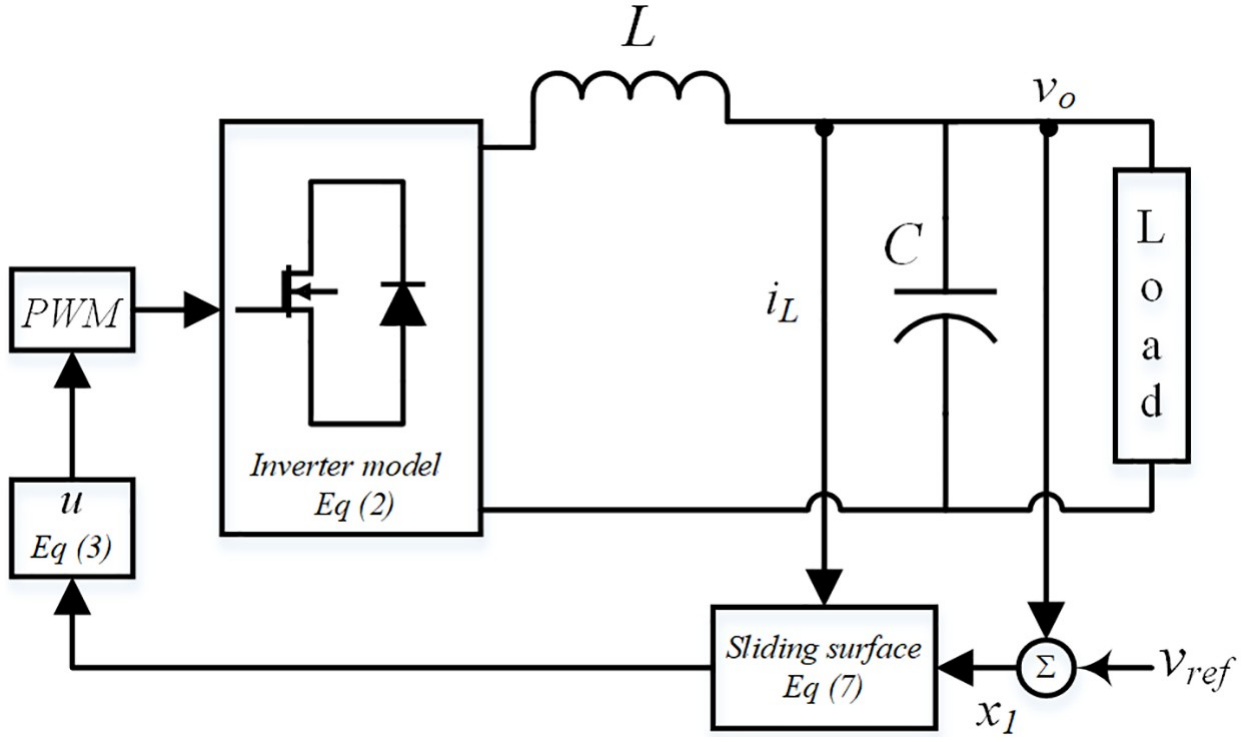
Sliding surface based upon state feedback.

**Fig 4:**
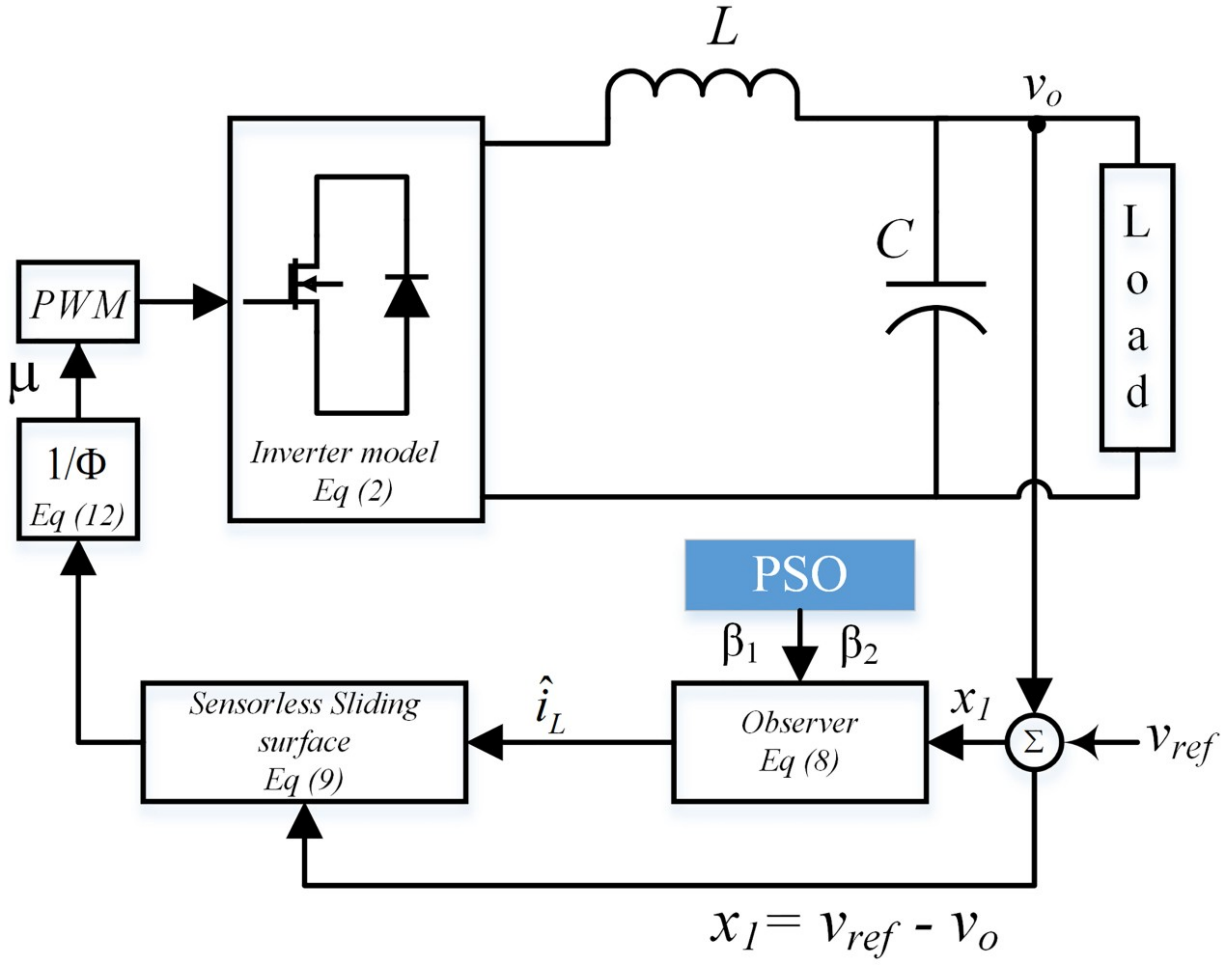
Proposed sensorless sliding mode control scheme with smoothed control law.

It is obvious from (7) that both of state variables are required for sliding surface design and illustrated in Fig 3. In order to remove the dependency on the current sensor, estimation of the inductor current is needed. For the system dynamics described in (2) the state observer presented in [30] can be adapted to be

$$\begin{array}{c} \left\{ \begin{array}{c} {\dot{\hat{v}}}_{o}=\frac{{\hat{i}}_{L}}{C}-\frac{v_{o}}{RC}+\beta_{1}\left(v_{o}-{\hat{v}}_{o}\right)\\ {\dot{\hat{i}}}_{L}=\frac{uV_{dc}}{L}-\frac{v_{o}}{L}+\beta_{2}\left(v_{o}-{\hat{v}}_{o}\right) \end{array}\right. \end{array}$$ (8)

where *β*_1_ and *β*_2_ are the positive observer constants to be designed and the observer gain selection is covered in section 4. Based on the observer proposed in (8) the sensorless sliding surface can be presented as

$$\begin{array}{c} \hat{s}\left(x\right)=\lambda \left(v_{ref}-v_{o}\right)-\frac{{\hat{i}}_{L}}{C}+\frac{v_{o}}{RC}+{\dot{v}}_{ref} \end{array}$$ (9)

where *v*_*o*_ is available for measurement while ${\hat{i}}_{L}$ is the output of the observer as described in Fig 4. Thus, the control law presented in (3) can be rewritten as

$$\begin{array}{c} u=sign(\hat{s}\left(x\right)) \end{array}$$ (10)

where *ŝ*(*x*) is the observer based sliding surface. The dynamic control law presented in (10) will tend to converge the system states to origin however the chattering effect due to the discontinuous sign(.) function is still present.

### 3.1 Boundary layer design for smooth control law

**Fig 5:**
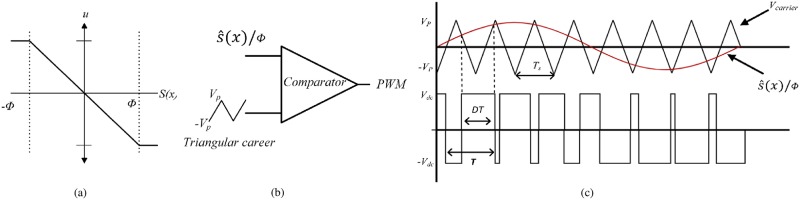
Smoothed control law using PWM (a) boundary layer (b) PWM generator (c) PWM output switching signal.

To eliminate the chattering phenomenon, boundary layer solution is proposed as shown in Fig 5a, in which the discontinuous control signal *u*(*u* = *sign*(*ŝ*(*x*))) is replaced by $\mu =-\frac{\hat{s}\left(x\right)}{\phi }$ where *ϕ* is the thickness of boundary layer. The selection of *ϕ* is a critical task as it provides a trade-off between the robustness of system performance and chattering reduction. It can be seen from Fig 5a that a large value of *ϕ* will attenuate the chattering but it would be done at the expense of system robustness. For inverter application, the smoothed control signal *μ* cannot be applied directly to the inverter and a PWM modulator is needed to generate the equivalent switching pulses. Thus, by considering PWM carrier and modulating signals frequency limitations, the boundary layer has been designed in relation to these signals that will preserve the robustness of the system while overcoming chattering.

The PWM output voltage contains the same fundamental frequency as the modulating signal. Thus, for proper PWM signal generation, the slope of the triangular carrier signal must be greater than the smoothed control law *μ*, fed as modulating signal to the PWM generator [31] as illustrated in Fig 5b. The slope of the triangular carrier signal can be defined as

$$\begin{array}{c} m_{t}=\frac{4V_{p}}{T_{s}} \end{array}$$ (11)

where *V*_*p*_ and *T*_*s*_ are the magnitude and switching time of the carrier signal, respectively. Then the input to the PWM generator can be represented as

$$\begin{array}{c} \mu =\frac{1}{\phi }\left(\lambda x_{1}+x_{2}\right) \end{array}$$ (12)

By inserting Eq (5) in (12), then

$$\begin{array}{c} \mu =\frac{1}{\phi }\left(\lambda \left(v_{ref}-v_{o}\right)+\left({\dot{v}}_{ref}-{\dot{v}}_{o}\right)\right) \end{array}$$ (13)

During steady-state condition, the error (*v*_*ref*_ − *v*_*o*_) → 0 as the inverter output is considered as pure sinusoidal, i.e. *v*_*o*_ = *v*_*ref*_. Thus (13) yields

$$\begin{array}{c} \mu =\frac{1}{\phi }\left({\dot{v}}_{ref}-{\dot{v}}_{o}\right) \end{array}$$ (14)

As ${\dot{v}}_{o}=\frac{1}{C}i_{c}$, Eq (14) can be represented as

$$\begin{array}{c} \mu =\frac{1}{\phi C}\left(i_{ref}-i_{C}\right) \end{array}$$ (15)

During the steady-state condition, the capacitor current error (*i*_*ref*_ − *i*_*C*_) can be viewed as inductor ripple current Δ*i*_*L*_ [32]. Thus, (15) can be defined as

$$\begin{array}{c} \mu =\frac{\Delta i_{L}}{\phi C} \end{array}$$ (16)

Also, it is known that

$$\begin{array}{c} \Delta i_{L}=\frac{V_{dc}-v_{o}}{L}DT \end{array}$$ (17)

where *D* and *T* are the duty cycle and time period of the PWM output as illustrated in Fig 5c. Thus, with *v*_*o*_ = *V*_*dc*_ × *D*, Eq (16) can be represented as

$$\begin{array}{c} \mu =\frac{V_{dc}\left(1-D\right)}{\phi CL}DT \end{array}$$ (18)

By taking the derivative of (18) w.r.t. *T* and selecting *D* = 50%, the slope of input signal is obtained as follows;

$$\begin{array}{c} m_{\mu }=\frac{V_{dc}}{4\phi LC} \end{array}$$ (19)

According to the limitation of PWM generator the slope of input signal must be less than the slope of carrier signal i.e.

$$\begin{array}{c} m_{t}\gg m_{\mu } \end{array}$$ (20)

Thus

$$\begin{array}{c} \frac{4V_{p}}{T_{s}}\gg \frac{V_{dc}}{4\phi LC} \end{array}$$ (21)

$$\begin{array}{c} \phi \gg \frac{0.0625V_{dc}}{LCV_{p}f_{s}} \end{array}$$ (22)

Eq (22) gives the minimum value of the boundary layer that needs to be fulfilled in order to reduce the chattering effect at *μ* while preserving the robustness of inverter performance. It should be noted that the derived boundary thickness depends only on the parameters of the system, regardless of the state variables. This continuous control signal will ensure fast dynamic response with minimal THD at the inverter output terminals as presented in section 6.

## 4 Stability analysis

This section illustrates the stability analysis of the proposed closed loop system. Firstly, the observer stability is ensured by proving that the error dynamics converge to zero and then it is proven that the system dynamics and sliding surface converge to zero in finite time. To meet this objective, the observation errors are defined as

$$\begin{array}{c} \left\{ \begin{array}{c} e_{1}=v_{o}-{\hat{v}}_{o}\\ e_{2}=i_{L}-{\hat{i}}_{L} \end{array}\right. \end{array}$$ (23)

By taking the derivative of (23) the error dynamics can be written as

$$\begin{array}{c} \left\{ \begin{array}{c} {\dot{e}}_{1}={\dot{v}}_{o}-{\dot{\hat{v}}}_{o}\\ {\dot{e}}_{2}={\dot{i}}_{L}-{\dot{\hat{i}}}_{L} \end{array}\right. \end{array}$$ (24)

and substituting the system dynamics (2) and observer dynamics (8) in (24), then

$$\begin{array}{c} \left\{ \begin{array}{c} {\dot{e}}_{1}=\frac{i_{L}}{C}-\frac{v_{o}}{RC}-\frac{{\hat{i}}_{L}}{C}+\frac{v_{o}}{RC}-\beta_{1}e_{1}\\ {\dot{e}}_{2}=\frac{uV_{dc}}{L}-\frac{v_{o}}{L}-\frac{uV_{dc}}{L}+\frac{v_{o}}{L}-\beta_{2}e_{1} \end{array}\right. \end{array}$$ (25)

Eq (25) is reduced to

$$\begin{array}{c} \left\{ \begin{array}{c} {\dot{e}}_{1}=-\beta_{1}e_{1}+\frac{1}{C}e_{2}\\ {\dot{e}}_{2}=-\beta_{2}e_{1} \end{array}\right. \end{array}$$ (26)

$$\begin{array}{c} \left[\begin{array}{c} {\dot{e}}_{1}\\ {\dot{e}}_{2} \end{array}]=[\begin{array}{cc} -\beta_{1} & \frac{1}{C}\\ -\beta_{2} & 0 \end{array}][\begin{array}{c} e_{1}\\ e_{2} \end{array}\right] \end{array}$$ (27)

which can be further written as

$$\begin{array}{c} \dot{e}=Ae \end{array}$$ (28)

By proper selection of the parameters *β*_1_ and *β*_2_, the characteristic polynomial for system (28) *P*(*s*) = |*sI* − *A*| is made Hurwitz stable which leads the estimation errors *e*_1_ and *e*_2_ converge to zero in a finite time. The switching control law $\mu =-\frac{\hat{s}\left(x\right)}{\phi }$ will force the system to move towards the sliding surface described in (9) i.e *ŝ*(*x*) = 0 and stays thereafter. Rewriting (9) as

$$\begin{array}{c} 0=\lambda x_{1}+{\dot{v}}_{ref}-\frac{{\hat{i}}_{L}}{C}+\frac{v_{o}}{RC} \end{array}$$ (29)

and by inserting the value of ${\dot{v}}_{ref}$ from (5) in (29), then

$$\begin{array}{c} 0=\lambda x_{1}+\left({\dot{x}}_{1}+{\dot{v}}_{o}\right)-\frac{{\hat{i}}_{L}}{C}+\frac{v_{o}}{RC} \end{array}$$ (30)

By using the output voltage dynamics from (2), then (30) becomes

$$\begin{array}{c} {\dot{x}}_{1}=-\lambda x_{1}-\frac{1}{C}e_{2} \end{array}$$ (31)

With the proper value of observer gains, the estimation error *e*_2_ → 0 in finite time, under the proposed control law, leaving the inverter error dynamics response as of first order *ẋ*_1_ = −λ*x*_1_ with time constant $\tau =\frac{1}{\lambda }$. Thus, the proper selection of λ > 0 will force the tracking error *x*_1_ converge to zero asymptotically. This completes the proof.

**Gain Tuning**: For the proposed control law only critical parameter that needs to be designed is λ. The convergence of error dynamics *ẋ*_1_ = −λ*x*_1_ can be written as *x*_1_ = *x*_1_(*t*_0_).*e*^−λ*t*^ thus, higher values of λ is preferred to achieve the faster response with minimum steady state error. But it is obvious that the response cannot be made faster than switching signal, therefore, λ <= *f*_*s*_ is selected.

## 5 Observer gain optimization using PSO

**Fig 6:**
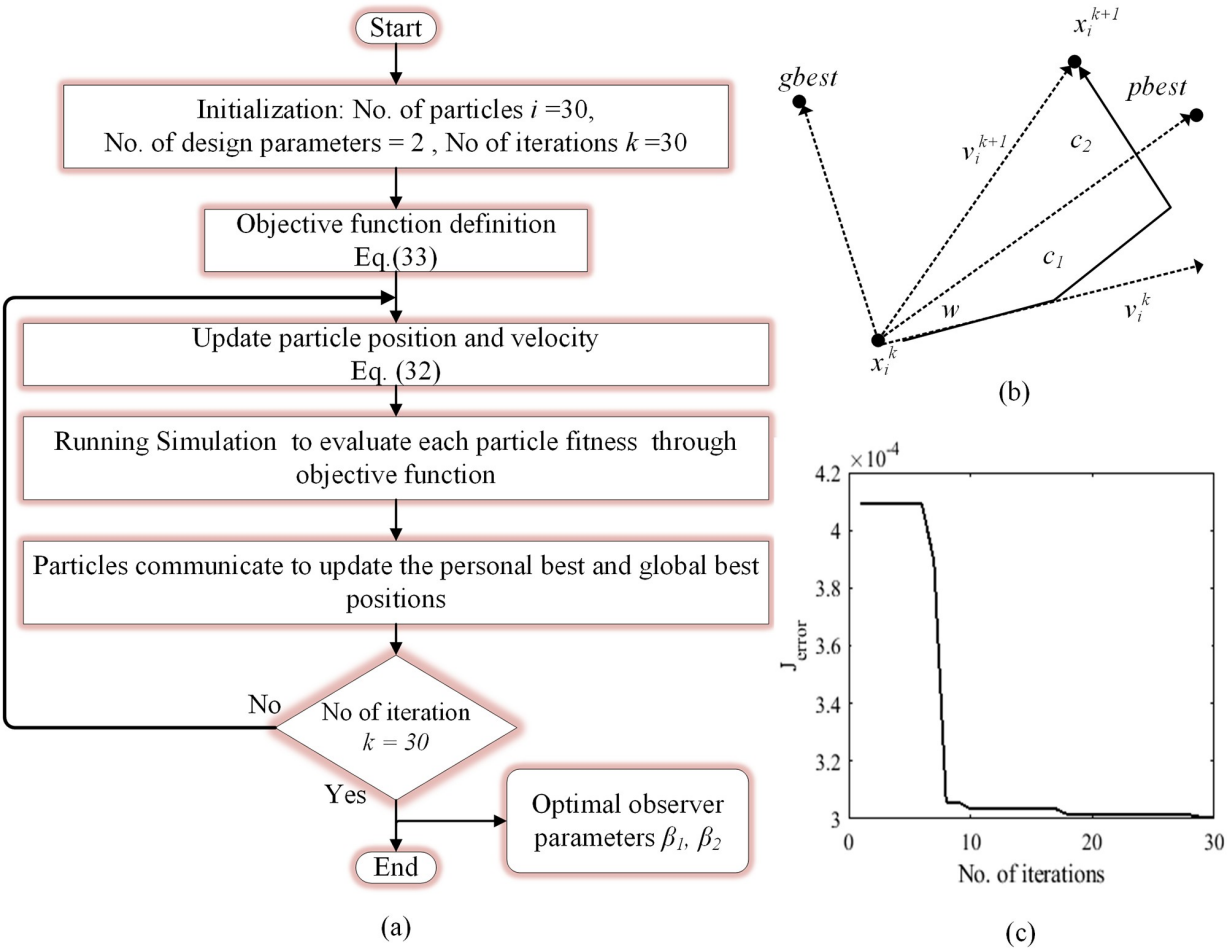
(a) Flow chart for PSO algorithm (b) position and velocity update of particles (c) convergence of PSO.

The heuristic approach to find the best values for observer gain is exhaustive and in many cases, it does not lead to an optimal solution. Also, for most observer design approaches, accurate mathematical system description is required that makes the gain derivation a challenging task. In this work, observer gains *β*_1_ and *β*_2_ in (8) are designed using offline particle swarm optimization (PSO) technique. The procedure of the PSO is presented in Fig 6a where the members of the swarm are represented as particles and each particle provide a potential solution to the estimation error-based objective function.

At each iteration, the specified objective function is evaluated for each particle and particles are updated with personal best and global best positions which indicate the best solution provided by individual particle and entire swarm, respectively. Before each iteration, particles change their position as follows;

$$\begin{array}{c} \left\{ \begin{array}{c} v_{i}^{k+1}=wv_{i}^{k}+c_{1}.rand_{1}\left(‥\right)\left(pbest_{i}-x_{i}^{k}\right)+c_{2}.rand_{2}\left(‥\right)\left(pbest_{i}-x_{i}^{k}\right)\\ x_{i}^{k+1}=x_{i}^{k}+v_{i}^{k+1} \end{array}\right. \end{array}$$ (32)

where $v_{i}^{k}$ is the velocity of *i*^*th*^ particle at *k*^*th*^ iteration, *w*, *c*_1_ and *c*_2_ are the weighting factors, $x_{i}^{k}$ is the current position of the particle *i* at *k*^*th*^ iteration, *pbest* and *gbest* are the optimal solutions given by particle *i* and entire swarm, respectively, as shown in Fig 6b. The values of parameters are listed in Table 1. The primary goal for this VSI system is to minimize the estimation errors *e*_1_ and *e*_2_ along with tracking error *x*_1_. For that purpose, the objective function to be evaluated by the swarm particles is specified as

$$\begin{array}{c} J=\int_{0}^{t}|e_{1}|dt+\int_{0}^{t}|e_{2}|dt+\int_{0}^{t}\left|x_{1}\right|dt \end{array}$$ (33)

**Fig 7:**
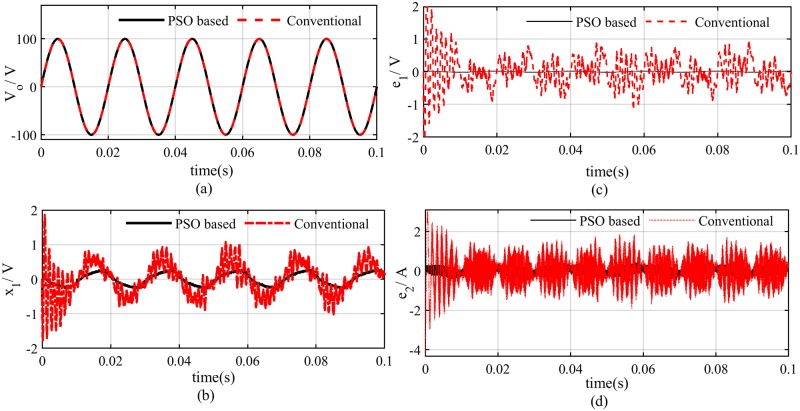
PSO based optimal control performance (a) output voltage (b)output voltage tracking error (c) voltage estimation error (d) current estimation error.

The PSO will minimize the objective function by selecting the optimal observer parameters *β*_1_ and *β*_2_ as shown in Fig 6c, where more accurate estimation of the inductor current ${\hat{i}}_{L}$ is achieved. A comparison of PSO based algorithm and heuristic-based gain selection is given in Fig 7 which clearly depicts that PSO based algorithm exhibits superior performance in terms of both tracking and estimation error minimization.

**Table 1:** PSO parameters.

symbol	Description	Values
*k*	No. of iteration	10
*i*	No. of particles	20
*c*_1_	weighting factor	1.42
*c*_2_	weighting factor	1.42
*w*	weighting factor	0.9

## 6 Simulation results and discussion

The proposed smooth dynamic SMC is validated through MATLAB Simulink tools. The performance of the proposed controller is compared with the PID controller tuned by using PSO to minimize the tracking error. The discrete components values used for simulation and designed controller parameters are presented in Tables 2 and 3, respectively. The performance comparison has been made under three cases i.e. under nominal load, sudden linear load variations and highly non-linear rectifier load.

**Table 2:** Characteristic values used for simulation.

Description	Symbol	Values
Input *DC* voltage	*V*_*dc*_	200 *V*
Reference Voltage	*v*_*ref*_	100 *sin*(2*πft*)*v*
Inductance	*L*	1 × 10^−3^ *H*
Capacitance	*C*	200 × 10^−6^ *F*
Nominal linear load	*R*	100Ω
Non-linear load	*R*_*s*_, *R*_*NL*_, *C*_*NL*_	0.32Ω, 18Ω, 3200*μF*

**Table 3:** Controller parameters.

Description	Symbol	Values
Observer gain	*β*_1_	474.94
Observer gain	*β*_2_	5000
Sliding constant	λ	15000
Boundary layer	*ϕ*	58020
PWM swithing frequency	*f*_*s*_	15000
Sampling time	*τ*	20 *μ* sec

### Case 1: Performance analysis at nominal load

**Fig 8:**
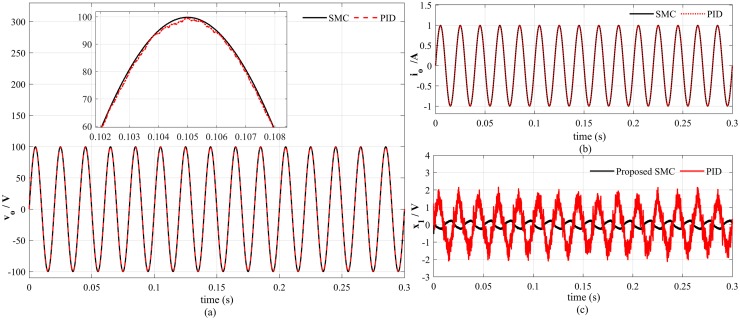
Performance at nominal load (a) output voltage (b) load current (c) voltage tracking error.

**Fig 9:**
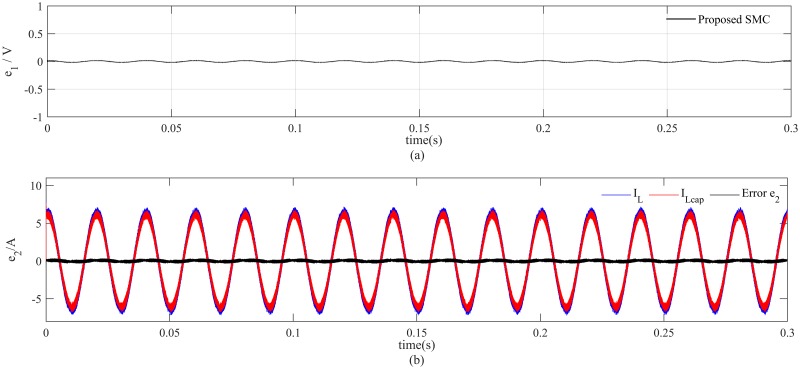
Observer performance at nominal load (a) voltage estimation error (b) current estimation error.

**Fig 10:**
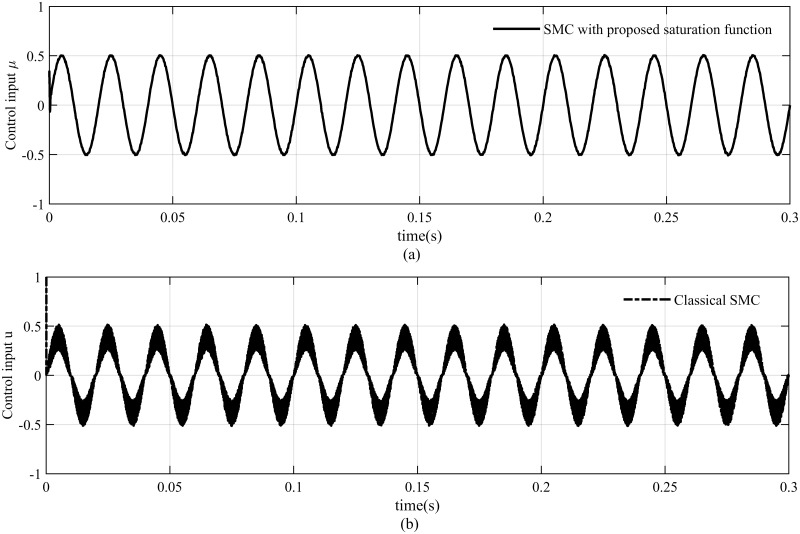
Control input (a) SMC with proposed saturation function (b) classical SMC.

At nominal load, the proposed controller can track the reference voltage more accurately and there is nominal distortion in the output voltage and load current as depicted in Fig 8a and 8b. It is clearly shown in Fig 8c that the tracking error for PID controller is 4*V*_*p*−*p*_ which is significantly greater than the tracking error 0.4*V*_*p*−*p*_ of the proposed method. The voltage and current estimation errors also converge to zero immediately as illustrated in Fig 9a and 9b, respectively, which proves the effectiveness of the proposed observer. In addition, simulation results also depicts that the proposed saturation function significantly attenuates the chattering effect in the control signal as presented in Fig 10.

### Case 2: Performance analysis under linear load variations

A step variation in the linear load has been made according to (34) where these resistive load variations occur on the system after the system has already reached the steady-state condition.

$$\begin{array}{c} \left\{ \begin{array}{c} R=100\;for\;t\leq 0.085\\ R=150\;for\;0.205>t\geq 0.085\\ R=50\;for\;t\geq 0.205 \end{array}\right. \end{array}$$ (34)

**Fig 11:**
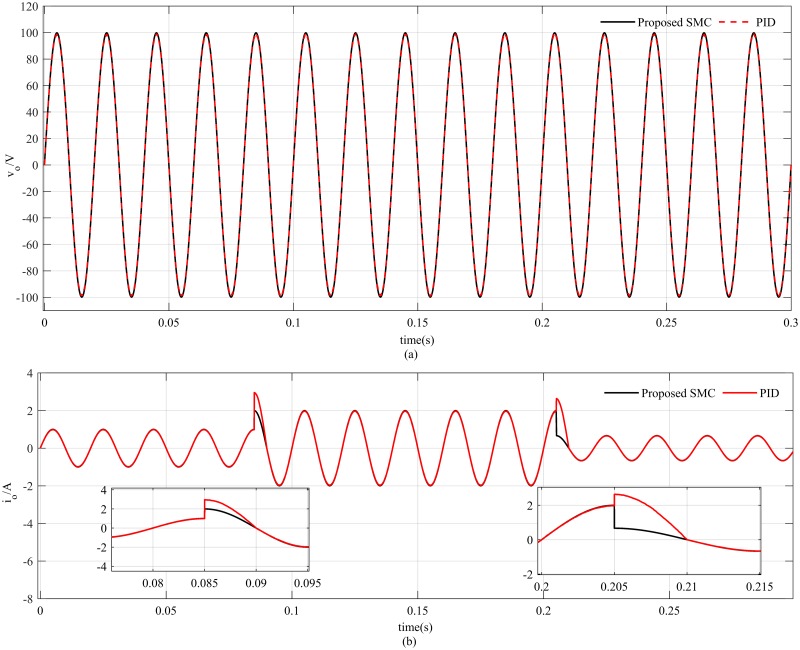
Reference tracking with linear load variations (a) output voltage (b) load current.

**Fig 12:**
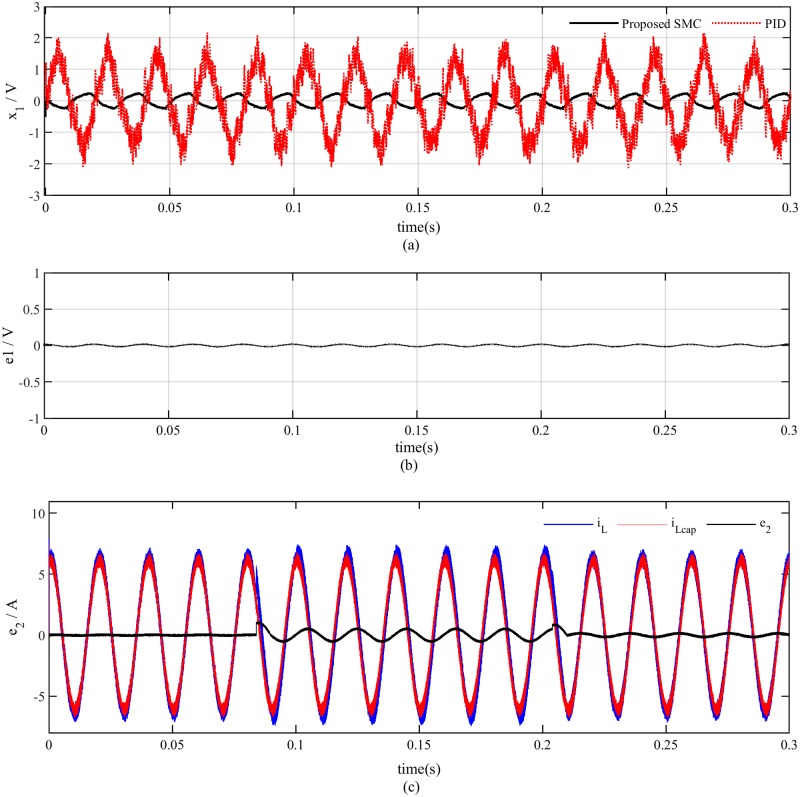
Reference tracking and observer performance (a) voltage tracking error (b) voltage estimation error (c) current estimation error.

**Fig 13:**
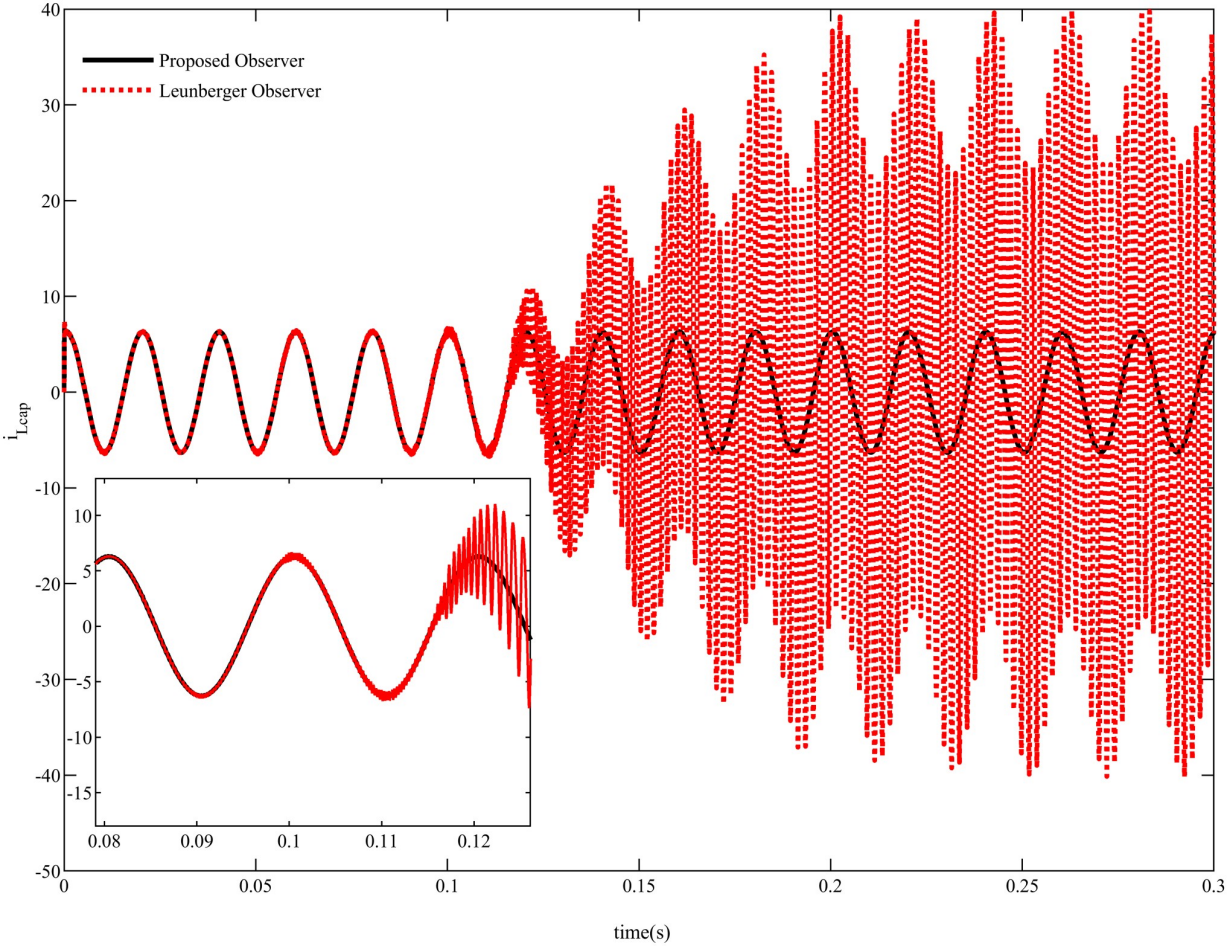
Observer performance comparison.

In this case, the proposed SMC exhibits robust performance in terms of both output voltage and load current as shown in Fig 11a and 11b but an overshoot of 0.5*A* is observed in the load current for PID controller for both increasing and decreasing load conditions. The proposed dynamic SMC exhibits superior performance in terms of tracking error which remains at 0.4*V*_*p*−*p*_ as compared to PID based system tracking error which is about 4*V*_*p*−*p*_ as illustrated in Fig 12a. The voltage estimation error also converges to zero in a robust manner as indicated in Fig 12b whereas the current estimation error remains within 0.5*A* due to variation of the load as depicted in Fig 12c. The proposed PSO based observer is compared with luenberger observer in Fig 13 which depicts that both the observer exhibits almost similar performance at nominal load. But after making load variation at 0.085sec the luenberger observer becomes unstable while the proposed observer shows consistent performance.

### Case 3: Performance analysis with non-linear load

**Fig 14:**
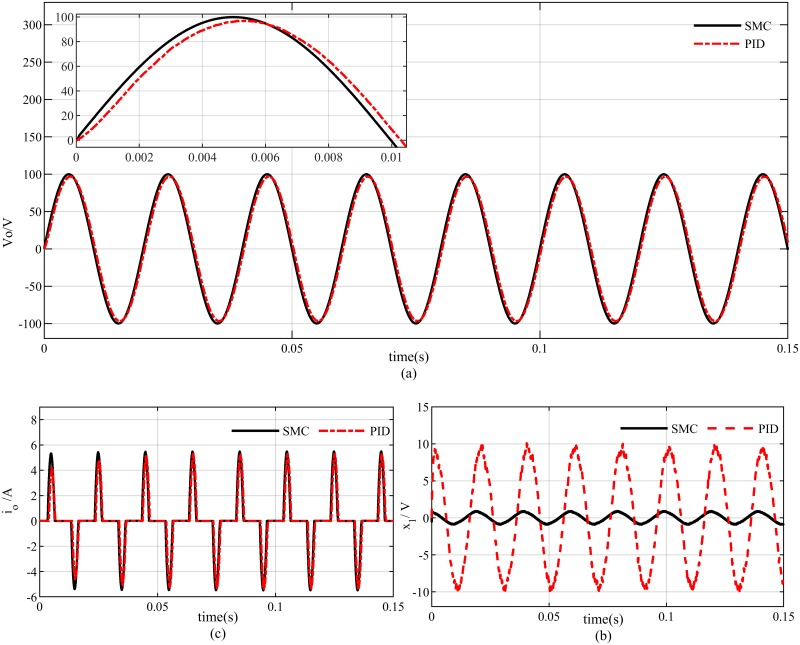
Reference tracking with non linear load (a) output voltage (b) tracking error (c) load current.

The most common non-linear load attached to the inverter output is full-wave bridge rectifier, that is widely used for battery charging purposes. Under this type of load, the proposed SMC algorithm exhibit good tracking performance as presented in Fig 14a. A phase shift in the output voltage has been observed in PID based system output which results in a tracking error of about 20*V*_*p*−*p*_ as compared to 0.4*V*_*p*−*p*_ of proposed SMC as presented in Fig 14b. Moreover, a settling time of 0.05s is needed for the load current to reach the steady-state in case of PID controller as illustrated in Fig 14c.

### Case 4: THD analysis

**Fig 15:**
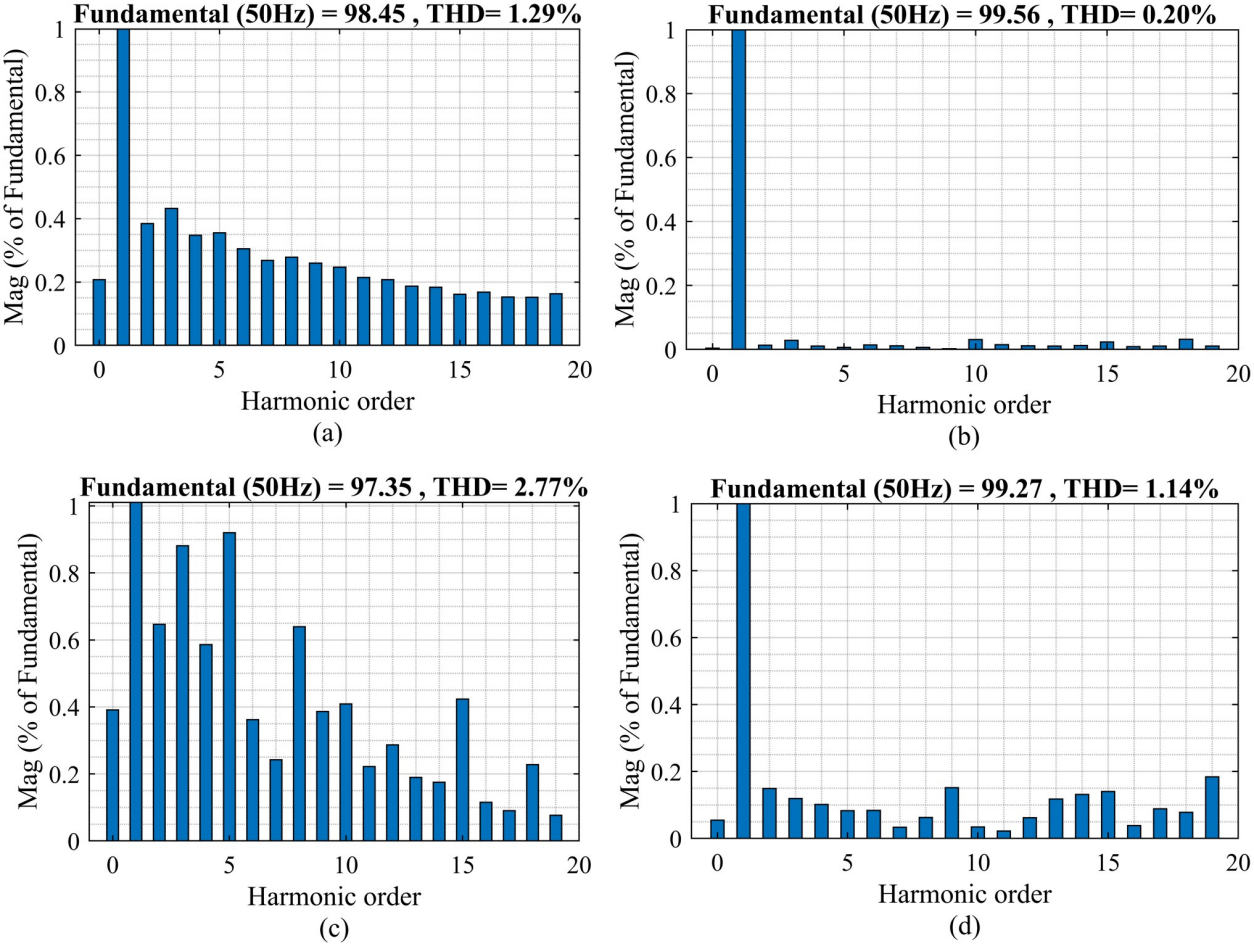
Total harmonic distortion for (a) PID against linear load (b) proposed SMC against linear load (c) PID against non-linear load (d) proposed SMC against non-linear load.

The novel boundary layer selection enables the proposed algorithm to exhibit superior performance in terms of harmonic rejection for both linear and non-linear loading conditions. For linear loads, the THD is 1.29% for PID while the proposed SMC limits the THD at 0.20% as described in Fig 15a and 15b, respectively. The proposed algorithm also performs well in terms of THD elimination at non-linear loads and contains the THD at 1.14% as compared to the PID controller with a THD of 2.77% as illustrated in Fig 15d and 15c.

Furthermore, a comparison of the proposed algorithm with the previous works is presented in Table 4 which reflects that the proposed controller exhibits superior performance compared to the previous works in terms of tracking error and THD for both linear and non-linear loads.

**Table 4:** Proposed SMC and its comparison with other works.

References	[2]	[14]	[15]	[33]	Proposed method
Controller Type	SMC	Repetitive	MPC	Multiloop *H*_∞_	Proposed method
Sensorless	No	No	No	No	Yes
Input *DC* voltage	390	400	295	180	200
Output *AC* voltage (rms)	110	110	110	110	70.7
Filter Capacitor (*μF*)	6.6	100	6.5	300	200
Filter Capacitor (*mH*)	0.880	3	10	1	1
Switching frequency *f*_*sw*_(*kHz*)	15	10	-	20	15
Voltage Tracking error (%)	2.7	4	0.8	1.5	0.4
THD linear load (%)	0.4	0.73	1.6	1	0.20
THD non-linear load (%)	1.70	1.52	2.6	12	1.14

## 7 Conclusion

In this paper, a smoothed SMC of single-phase VSI with inductor current observer has been presented for voltage reference tracking and THD removal against linear and non-linear load disturbances. By considering PWM operating limitations, a smoothed control law has been designed that significantly reduces the chattering effect and removes the unwanted oscillations from the inverter output. A modified high gain state observer has been proposed to estimate the inductor current in the presence of load disturbances while the complexity of optimal gain design has been removed by using the PSO technique. The simulation results and the comparative analysis of the proposed algorithm with its counterparts proved the superiority of the proposed algorithm in terms of output voltage tracking error and THD reduction for all loading conditions.
